# CAIX-targeting radiotracers for hypoxia imaging in head and neck cancer models

**DOI:** 10.1038/s41598-019-54824-5

**Published:** 2019-12-11

**Authors:** Fokko J. Huizing, Javad Garousi, Jasper Lok, Gerben Franssen, Bianca A. W. Hoeben, Fredrik Y. Frejd, Otto C. Boerman, Johan Bussink, Vladimir Tolmachev, Sandra Heskamp

**Affiliations:** 1 Departments of Radiation Oncology, Radboud University Medical Center, Nijmegen, The Netherlands; 2 Department of Immunology, Genetics and Pathology, Uppsala University, Uppsala, Sweden; 3 Departments of Radiology and Nuclear Medicine, Radboud University Medical Center, Nijmegen, The Netherlands; 4 Affibody AB, Solna, Sweden

**Keywords:** Cancer imaging, Predictive markers

## Abstract

Hypoxia-induced carbonic anhydrase IX (CAIX) expression is a prognostic marker in solid tumors. In recent years many radiotracers have been developed, but a fair comparison of these compounds is not possible because of the diversity in tumor models and other experimental parameters. In this study we performed a direct *in vivo* comparison of three promising CAIX targeting radiotracers in xenografted head and neck cancer models. The biodistribution of [^111^In]In-DOTA-ZCAIX:2 was directly compared with [^111^In]In-DTPA-G250-F(ab′)_2_ and [^111^In] In-DTPA-G250 in female BALB/C nu/nu mice bearing two HNSCC xenografts with different levels of CAIX expression. *In vivo* biodistribution was quantified by means of microSPECT/CT scans and *ex vivo* biodistribution was determined with the use of a γ-counter. Tumors were snap frozen and sections were stained for CAIX expression, vessels, hypoxia (pimonidazole) and tumor blood perfusion. Tracer uptake was significantly higher in SSCNij153 tumors compared to SCCNij185 tumors for [^111^In]In-DOTA-HE3-ZCAIX:2: 0.32 ± 0.03 versus 0.18 ± 0.01%ID/g,(p = 0.003) 4 h p.i., for [^111^In]In-DTPA-girentuximab-F(ab′)_2_: 3.0 ± 0.5%ID/g and 1.2 ± 0.1%ID/g (p = 0.03), 24 h p.i. and for [^111^In]In-DTPA-girentuximab: 30 ± 2.1%ID/g and 7.0 ± 1.0%ID/g (p = 0.0002) 72 h p.i. SPECT imaging with both [^111^In]In-DTPA-girentuximab-F(ab′)_2_ and [^111^In]In-DTPA-girentuximab showed a clear difference in tracer distribution between the two tumor models. The whole IgG, i.e. [^111^In]In-DTPA-girentuximab, showed the highest tumor-to-muscle ratio. We showed that different CAIX-targeting radiotracers can discriminate a low CAIX-expressing tumor from a high CAIX-expressing head and neck cancer xenografts model. In these hypoxic head and neck xenograft models [^111^In]In-DTPA-girentuximab showed the most promising results.

## Introduction

In oncology, molecular imaging is a rapidly growing field due to its potential to personalize treatment. Clinical studies have demonstrated that CAIX is a prognostic biomarker in almost all solid tumor types and its expression is associated with therapy resistance. Therefore, CAIX is one of the imaging targets of interest^1,2^.

The tumor microenvironment (TME) plays a crucial role in chemo-, radio- and immunotherapy. From a multitude of environmental factors, hypoxia and acidosis appear to be dominant in determining therapy resistance^3,4^. Tumor vasculature almost always develops in an irregular fashion, resulting in tumor regions that are deprived from sufficient blood supply and thus lack supply of sufficient oxygen and nutrients. In these hypoxic regions, cells have to adapt in order to survive. They undergo metabolic changes as they switch to anaerobic energy production, thereby producing more carbon dioxide_,_ lactate and protons, which generate a lower extracellular pH^5^. In this increased acidic TME, immune activity is suppressed and extracellular matrix is catabolized, resulting in migration of tumor cells^6^. Furthermore, the lack of oxygen is a well-known cause for radioresistance. Oxygen molecules are essential for the effectiveness of radiotherapy, possibly explained by the oxygen fixation hypothesis, which states that DNA-damage is chemically fixated by oxygen molecules. Hypoxic cells can be up to 3 times more resistant to radiotherapy than normoxic cells^7,8^. Overall, tumor hypoxia leads to more aggressive and therapeutic resistant tumor cells^9^.

Carbonic anhydrase IX (CAIX) is a hypoxia-related enzyme expressed on the membrane of cancer cells. Expression of CAIX is regulated by the transcription factor hypoxia inducible factor-1alpha (HIF-1α), and it catalyzes the conversion of carbon dioxide to bicarbonate and hydrogen ions by the extracellular domain. Furthermore, it interacts with lactate pumps and bicarbonate transporters, thereby regulating the internal and external pH in tumors^10,11^. Therefore, CAIX plays a key role in the adaptation of tumor cells to hypoxia.

In normal tissue, CAIX expression is absent, with the exception of the gastro-intestinal tract, where CAIX is expressed at low levels by enterocytes on the basolateral membranes. Under hypoxic conditions, tumor cells upregulate CAIX in a HIF-1α dependent manner. Under normoxic conditions HIF-1α is degraded rapidly by multiple proteins, including the Von Hippel-Lindau (VHL) protein. However, under low oxygen conditions, HIF-1α is stabilized and complexes with HIF-1β to form HIF-1, which regulates the transcription of different genes, including CAIX. A well-known mechanism of ubiquitous CAIX upregulation is a mutation in the VHL, which is present in the majority of renal cell carcinomas (RCC). This mutation prevents degradation of HIF-1α, resulting in HIF-1α accumulation and subsequent upregulation of CAIX. It is import to distinguish tumors with hypoxia-induced CAIX from tumors with VHL mutations, as the expression level and the number CAIX positive cells are much higher in tumors with a VHL mutation, and the expression is independent of hypoxia. For example, solid tumors without VHL mutation contain 0–60% hypoxic tumor areas with CAIX expression -as assessed immunohistochemically- whereas tumors with the VHL-mutation show CAIX expression above 85% on average^12,13^.

Detecting CAIX with a non-invasive imaging technique can be used to identify hypoxic and therapy resistant tumor areas in solid tumors in patients. This information is of great value to select the most appropriate treatment approach, such as CAIX-targeting drugs, hypoxia targeting drugs, optimal radiotherapy regimen and immunotherapy^14,15^.

In recent years, an increasing number of CAIX-targeting radiotracers have been developed and evaluated. First, the anti-CAIX monoclonal antibody G250 was successfully tested to image VHL-mutation-induced CAIX expression of renal cell carcinoma in 1993^16,17^. Further developments based on this monoclonal antibody led to a chimeric variant (girentuximab or cG250). However, complete monoclonal antibodies have a slow blood clearance, which results in high background and low tumor-to-normal tissue contrast at early time points post injection. To address this shortcoming, girentuximab antibody fragments, Fab′ and F(ab′)_2_, were produced and evaluated in renal cell carcinoma, but also in hypoxic tumors, including head and neck carcinoma and colorectal carcinoma xenografts. These fragments show a much faster blood clearance, but also a lower absolute tumor tracer uptake. Successful imaging with radiolabeled girentuximab-F(ab′)_2_ is possible as early as 24 hours post tracer injection^18,19^. In recent years other approaches have proven to be successful as well^20^, including application of engineered molecules, called affibody molecules. Affibody molecules are small (approximately 7 kDa) affinity proteins based on non-immunoglobulin scaffold^21^. Small size and high affinity to molecular targets makes affibody molecules suitable for the use as targeting vectors for radionuclide imaging^22^. These molecules are much smaller than the F(ab′)_2_ fragments (110 kDa) and can reach high affinity for CAIX, typically in the low nanomolar range. Garousi *et al*. developed several of these affibody molecules and tested them in an *in vivo* renal cell carcinoma model with outstanding results. The most promising affibody (ZCAIX:2) shows high tumor uptake with a very low blood and muscle uptake already at 4 hours post tracer injection^23^. The optimized variant, DOTA-HE_3_-ZCAIX:2, contains a histidine-glutamate-histidine-glutamate-histidine-glutamate (HE_3_) tag at N-terminus to minimize off-target interactions^24^. A maleimido derivative of a macrocyclic DOTA chelator was site-specifically conjugated to the C-terminal cysteine residue. Unlike acyclic chelators, DOTA enables stable labeling of this variant with a variety of radionuclides, including ^111^In for SPECT and ^68^Ga for PET imaging.

Multiple tracers have been tested for the purpose of CAIX imaging, both as a marker for CAIX-expressing clear cell renal cell carcinomas (ccRCC), as well as for hypoxia imaging. However, selecting the optimal tracer to image hypoxia-induced CAIX based only on literature data is difficult if not impossible. Previous studies have used varying experimental factors such as tumor model, time points, protein dose, etc. Therefore, a head-to-head comparison of the most promising tracers for imaging of hypoxia related expression of CAIX is warranted. For this reason, we evaluated the affibody ZCAIX:2, the antibody fragment girentuximab-F(ab′)_2_ and the complete antibody-based tracer in different head and neck xenograft models.

## Results

### Biodistribution

Tumor and normal tissue uptake of [^111^In]In-DOTA-HE_3_-ZCAIX:2, [^111^In]In-DTPA-girentuximab-F(ab′)_2_ and [^111^In]In-DTPA-girentuximab was determined in both SCCNij153 (high CAIX expression) and SCCNij185 (low CAIX expression) human head and neck tumor xenografts at time points reported to be optimal for each construct. At 4 hours post tracer injection, tumor uptake of [^111^In]In-DOTA-HE_3_-ZCAIX:2 was significantly higher in SSCNij153 tumors compared to SCCNij185 tumors (0.32 ± 0.03 versus 0.18 ± 0.01%ID/g, p = 0.003), resulting in average tumor-to-muscle ratios of 3.3 ± 1.5 and 1.3 ± 0.8 (p = 0.06). Normal tissue uptake was on average below 0.5%ID/g, except for the kidneys (190 ± 27%ID/g and 188 ± 17%ID/g in the SCCNij153 and SCCNij185 model, respectively).

[^111^In]In-DTPA-girentuximab-F(ab′)_2_ tumor uptake was 3.0 ± 0.5%ID/g and 1.2 ± 0.1%ID/g in the SCCNij153 and SCCNij185 models (p = 0.03), respectively, at 24 hours post tracer injection. Tumor-to-muscle ratios for the high CAIX model (SCCNij153) were 16 ± 12 and 3.7 ± 2.1 (p = 0.16). Normal tissue uptake was highest in the kidneys, spleen and liver: 71 ± 5, 6.9 ± 2.1 and 4.7 ± 1.2%ID/g in the SCCNij153 model.

**Figure 1:**
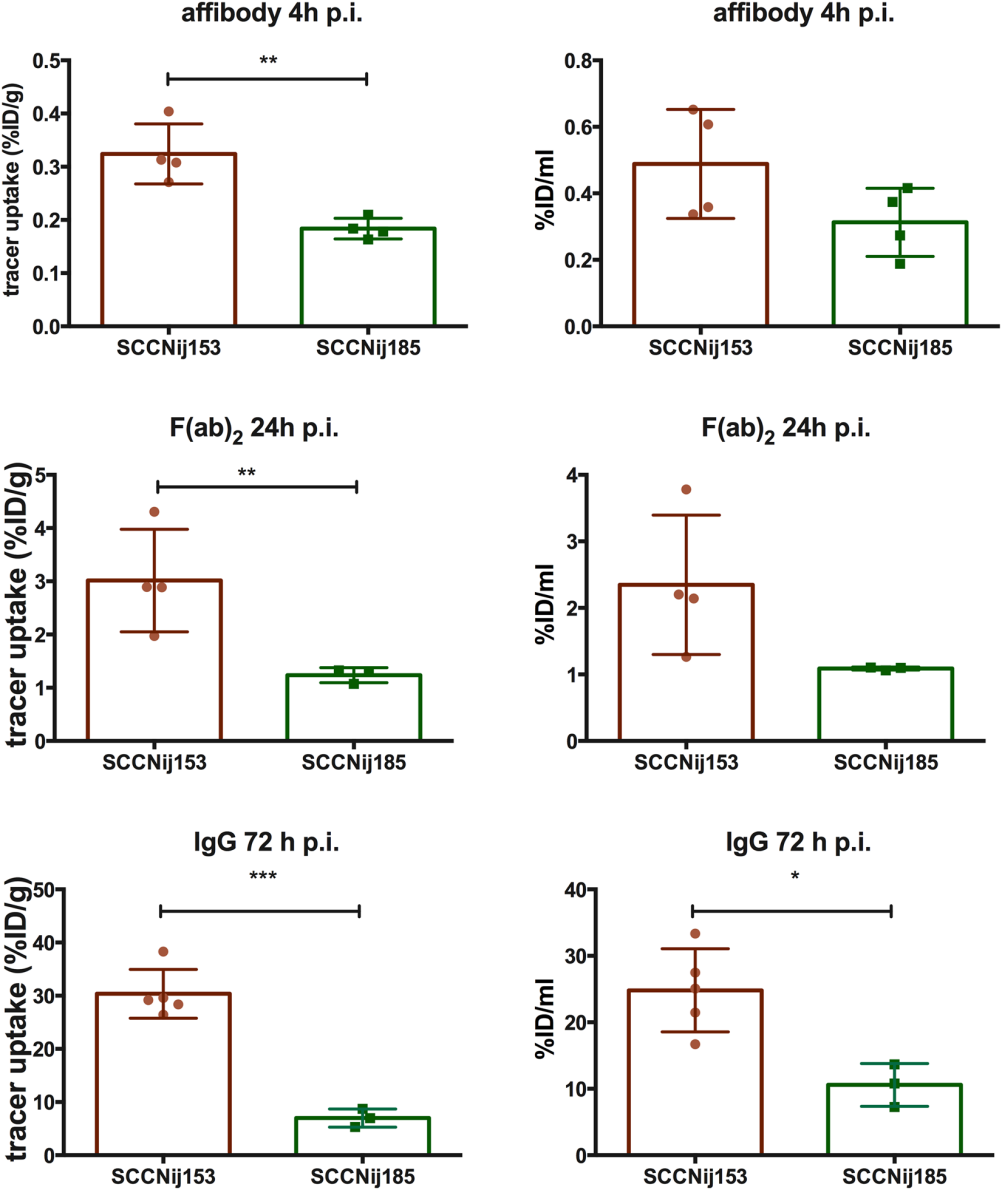
Tracer uptake (%ID/g) as determined *ex vivo* (**A**) and as derived from SPECT images (%ID/ml) (**B**) in the SCCNij153 (high CAIX-expressing) model and the SCCNij185 (low CAIX-expressing model). *p < 0.05;**p < 0.01; ***p < 0.001.

**Figure 2:**
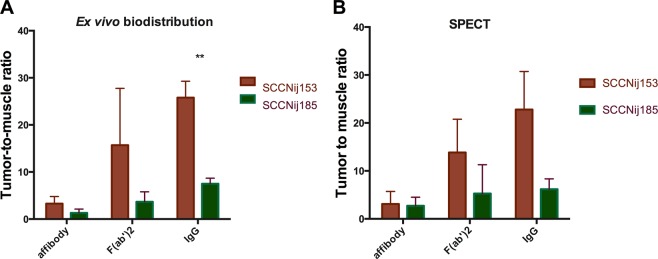
Tumor-to-muscle ratios calculated from *ex vivo* biodistribution measurements (**A**) and calculated from quantified SPECT measurements (**B**).

Highest tumor uptake was observed for [^111^In]In-DTPA-girentuximab: 30 ± 2.1%ID/g and 7.0 ± 1.0%ID/g in the SCCNij153 and SCCNij185 models (p = 0.0002), respectively, at 72 hours post tracer injection. This resulted in a tumor-to-muscle ratio of 26 ± 3.5 and 7.5 ± 1.1 for SCCNij153 and SCCNij185, respectively (p = 0.001). Normal tissue uptake values were below 8.0%ID/g, with 12.6% ± 3.8%ID/g still present in the blood. (Figs. 1 and 2A)

### MicroSPECT/CT imaging

**Figure 3:**
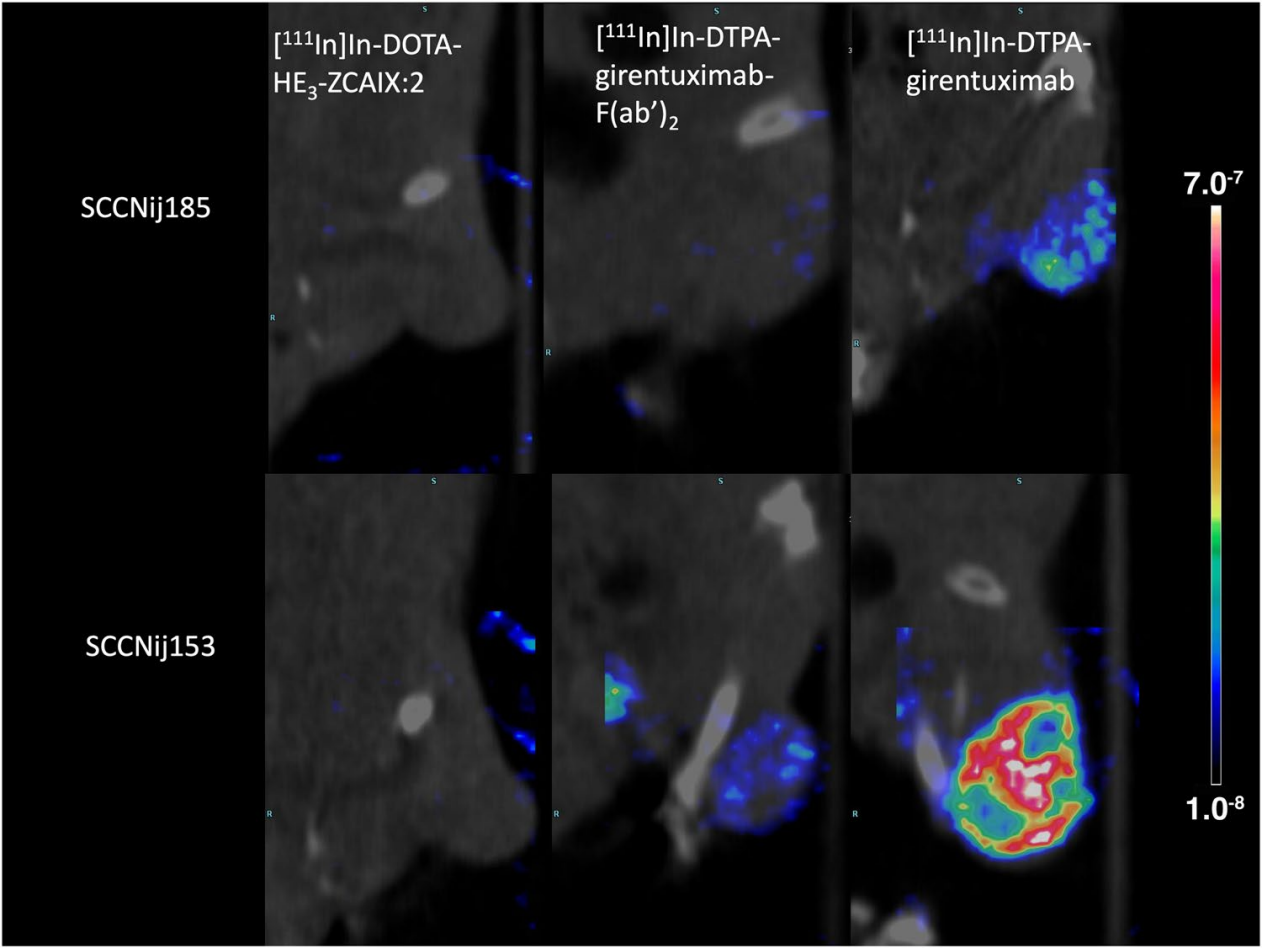
Examples of microSPECT/CT images of the right hind leg (tumor region) of the mice. For both HNSCC models, SCCNij153 and SCCNij185, images on the left were acquired 4 h post [^111^In]In-DOTA-HE_3_-ZCAIX:2 administration, images in the middle were acquired 24 h post [^111^In]In-DTPA-girentuximab-F(ab′)_2_ administration and images on the right 72 h post [^111^In]In-DTPA-girentuximab administration.

MicroSPECT images showed a higher concentration of [^111^In]In-DTPA-girentuximab-F(ab′)_2_ and [^111^In]In-DTPA-girentuximab in (high CAIX-expressing) SCCNij153 tumors than in (low CAIX-expressing) SCCNij185 tumors (Fig. 3). Quantitative analysis showed that tumor tracer uptake per volume (%ID/ml) was in agreement with the *ex vivo* biodistribution results. The tumor uptake and tumor-to-muscle ratios for the affibody-based tracer, the F(ab′)_2_-based and the whole antibody tracer were higher in SCCNij153 than in SCCNij185. (Figs. 1B and 2B)

### Immunohistochemistry

**Figure 4:**
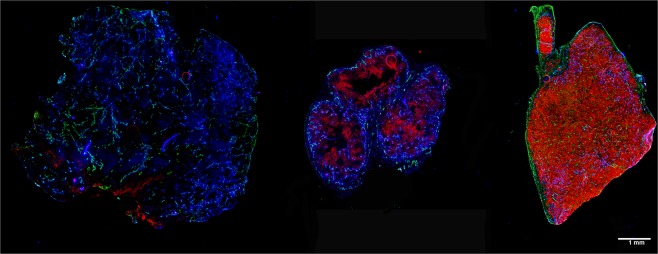
Example sections of 3 different tumor models. From left to right: SCCNij185, SCCNIj153 and SK-RC-52. Stained for CAIX (red), perfusion (blue) and vessels (green). Scalebar is 1000 µm.

SCCNij153 showed significantly higher CAIX expression than SCCNij185; tumor sections showed an antigen-positive fraction of 0.11 ± 0.02 versus 0.02 ± 0.01 (p < 0.01), respectively. As a reference, the SK-RC-52 renal cell carcinoma model, which constitutively overexpresses CAIX due to the VHL mutation, had a much higher CAIX positive fraction of 0.59 ± 0.15 (compared to SCCNij153 p = 0.03) (Fig. 4). Tumor tracer uptake of all three tracers correlated positively with the positive fraction for CAIX, with an R of 0.42 (p = 0.29), 0.91 (p = 0.01), and 0.32 (p = 0.48), respectively, for affibody-based, F(ab′)_2_-fragment based and whole antibody-based tracer. SCCNij153 was significantly more hypoxic than SCCNij185, with pimonidazole-positive fraction of 0.17 ± 0.03 versus 0.67 ± 0.02 (p < 0.01). SK-RC-52 sections were negative for pimonidazole, thus non-hypoxic.

### Intratumor correlation of CAIX expression and tracer uptake

**Figure 5:**
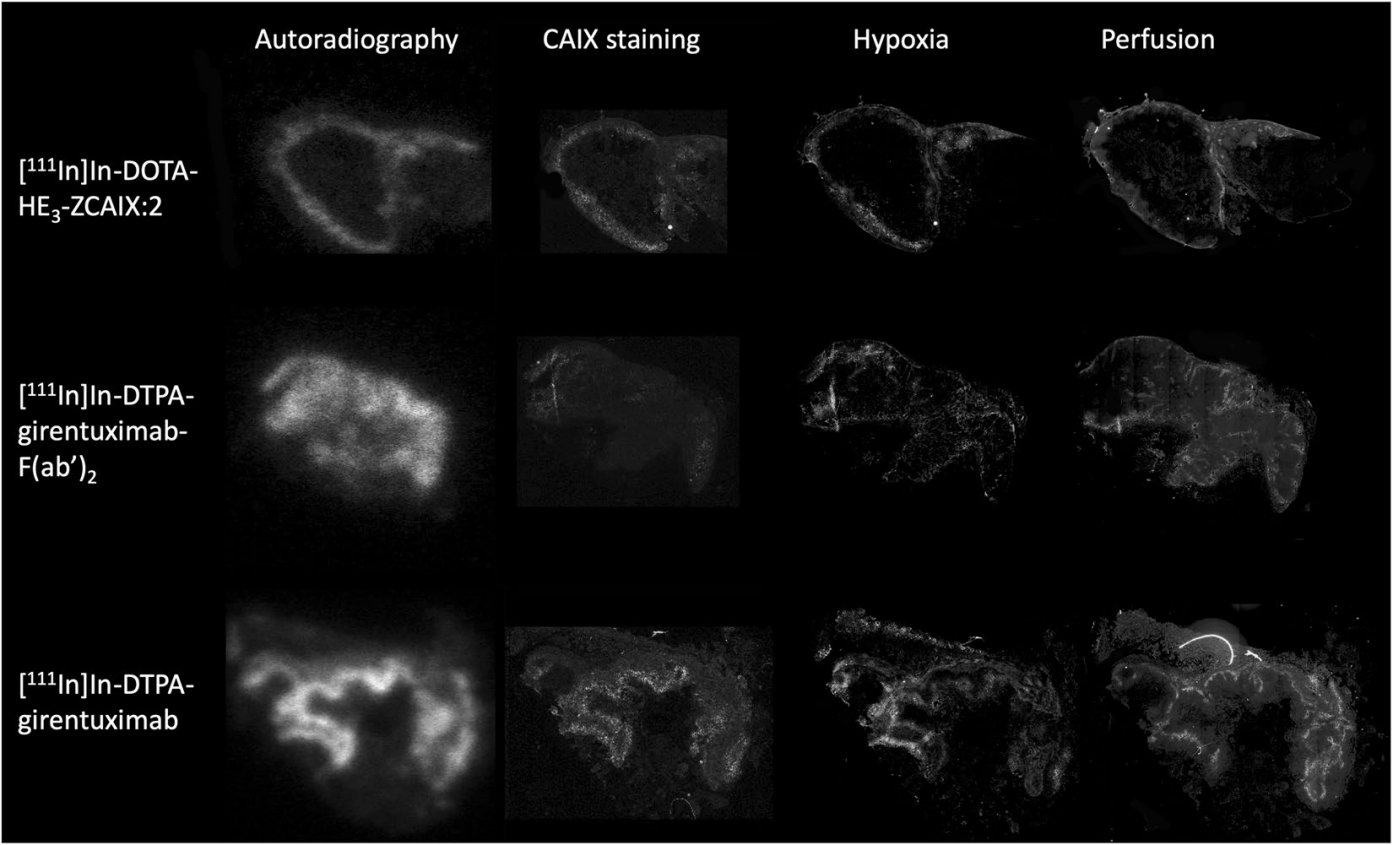
Examples of tumor sections of the SCCNij153 model. The left column shows autoradiography images. The same tumor sections were stained for CAIX expression (the second column), hypoxia (pimonidazole) (third column) and perfusion (Hoechst) (the right column).

Autoradiography of SCCNij153 showed heterogeneous tracer uptake for all three radiotracers (Fig. 5). Colocalization analysis per tracer group showed a positive correlation between the spatial distribution of tracer uptake and CAIX expression for the affibody-based, F(ab′)_2_-fragment based and whole antibody-based tracer, with a mean R of 0.25 ± 0.28 (p = 0.17), 0.19 ± 0.22 (p = 0.39) and 0.61 ± 0.15 (p = 0.001), respectively. One sample t-test showed only for the whole antibody-based tracer group a significant positive correlation.

## Discussion

Several new CAIX imaging agents have been developed and characterized in different tumor models in recent years. However, so far, no head-to-head comparison of these tracers for hypoxia-induced CAIX imaging has been performed. In the review of Lau *et al*. multiple CAIX-targeting tracers with very high tumor uptake levels and tumor-to-muscle ratios were discussed^20^. The highest ratios were found in experiments in mice with ccRCC xenografts, but these tracers were not tested in a hypoxia driven model^19,24–28^. One of the remaining questions is which tracer is most promising for the discrimination of low CAIX expressing tumors from high CAIX expressing tumors. Here we compared three promising CAIX-targeting radiotracers at optimal time points in two head and neck xenograft models that upregulate CAIX under hypoxic conditions in order to assess their discriminating capabilities. The affibody based, F(ab′)_2_ fragment based and intact IgG based tracers all were able to distinguish the high CAIX-expressing tumor model from the low CAIX-expressing tumor model. The intact IgG based tracer showed the highest contrast between these two tumor models and resulted in the highest tumor-to-muscle ratios.

The amount of tumor tracer uptake differs roughly a factor 10 between the affibody based, F(ab′)_2_ fragment based tracer and also between F(ab′)_2_ fragment based and whole antibody based tracer. The primary cause of these large gaps in accumulation is most likely the differences in blood circulation time of the tracers. The longer a compound is present in the blood the more time it has to penetrate and accumulate in the tumor. On the other hand, for imaging purposes low blood levels of radiotracer are needed at the time of scanning to create a contrast between tumor and background.

Although the tumor-to-muscle ratio of [^111^In]In-DOTA-HE_3_-ZCAIX:2 is approximately 4, the low absolute amount of tumor uptake limits the quality of the microSPECT images. The *ex vivo* biodistribution demonstrated that the uptake of [^111^In]In-DOTA-HE_3_-ZCAIX:2 was significantly higher in high CAIX expressing xenografts than in low CAIX expression xenografts. However, with an average tumor uptake of only 0.3%ID/g, it was difficult to visualize and quantify uptake by SPECT imaging, using 10 MBq of radiotracer and a scan time of 45 minutes. For F(ab′)_2_, we were able to visualize SSCNij153 tumors, which is in line with our previous results^19^. With the IgG based tracer, tumor uptake was the highest and we were able to image the tumor most clearly. High liver uptake with in IgG based tracers or kidney uptake with the affibody and F(ab′)_2_ based tracers could interfere with the visualization of CAIX expression in tumors located in the abdomen.

In a related study we assessed [^111^In]In-DOTA-HE_3_-ZCAIX:2 and F(ab′)_2_ in the non-hypoxic renal cell carcinoma model (SK-RC-52) which constitutively overexpresses CAIX because of a VHL mutation. In these experiments, affinity constants were determined: 1.2 nM for ^111^In]In-DOTA-HE_3_-ZCAIX:2 and 0.12 nM for [^111^In]In-DTPA-girentuximab-F(ab′)_2_^24^. The *in vivo* experiments in this study showed great similarities for normal tissue distribution, but a large difference in tumor tracer uptake compared to the SCCNij185 and SCCNij153 models. In the SK-RC-52 model, the tumor uptake of the affibody based tracer at 4 h and F(ab′)_2_-based tracer at 24 h p.i. was 15 ± 3%ID/g and 5 ± 1%ID/g, respectively. The approximately 50-fold higher tracer uptake of the affibody in the ccRCC model compared with the head and neck carcinoma models might be due to the fact that the level of CAIX expression in the SCCNij153 model is appreciably lower than the level of CAIX expression in SK-RC-52. In our immunohistochemical analysis we found an approximately 5 times higher fraction of CAIX-positive cells in SK-RC52 tumors than in SSCNij153. Unfortunately, we have no data on the number of receptors per cell in these models. Therefore, the difference in available CAIX proteins may be even larger. Our previous studies demonstrated that affibody molecules, with affinity in the low nanomolar range, were suitable for imaging of expression on the level of several hundred thousand antigens per cell, but subnanomolar affinities are required for imaging of lower expression levels^29^. Apparently, affinity of [^111^In]In-DOTA-HE_3_-ZCAIX:2 is good enough for imaging of CAIX in ccRCC with stable high ubiquitous expression, but might not be sufficient for imaging of low (and more dispersed) expression levels induced by hypoxia.

Earlier studies showed faster extravasation and faster diffusion through the interstitium for smaller molecules and thereby a deeper penetration into the tumor, resulting in a relatively high tracer accumulation^30^. We expected this mechanism to be beneficial for [^111^In]In-DOTA-HE_3_-ZCAIX:2 uptake and hypothesized that this would result in a higher tracer uptake in a poorly perfused tumor. The SK-RC-52 tumor is very well perfused, compared to both the SCCNij185 and SCCNij153 tumor (Fig. 4). Possibly, the fast clearance of the affibody based tracer counteracts the advantages of the affibody based tracer by not allowing sufficient time to diffuse through the tumor tissue^31^. Hence, tumor perfusion might have a more pronounced influence on radiotracer accumulation with a relative short half-life compared to those with a longer half-life.

Our autoradiography images showed a distinct ribbon- or band-like tracer distribution pattern, as observed in our earlier studies. The autoradiography signal positively co-localizes with the CAIX expression pattern in SCCNij153^32^ and thereby showed specificity of the tracers. Derived correlation coefficients from this co-localization did not differ significantly per tracer.

Furthermore, the staining of CAIX does only partly overlap with the hypoxia staining (pimonidazole), which was also seen in several other studies^33,34^. CAIX expression is the result of adaptation to hypoxia and therefore takes time^35^. As a result, CAIX-targeting tracers will not visualize acute hypoxia, but will visualize the possibly more relevant chronic hypoxia. Due to a mechanism of entrapment, traditional hypoxia tracers such as [^18^F]FMISO) and [^18^F]FAZA, will accumulate in tissue where the oxygen level is below 10 mmHg at any point during circulation of the compound.

An optimal tracer for CAIX imaging in the clinic should preferably allow imaging at early time points post tracer injection, as this is most practical for patients and it opens opportunities for repeated imaging to monitor therapy response. Therefore, rapid tumor accumulation and blood clearance is preferable. From this perspective, affibody molecules seem ideal candidates as they are known for their rapidly accumulation in tumor tissue and clearance from non-target tissues. Our previous study has demonstrated that [^111^In]In-DOTA-HE_3_-ZCAIX:2 seems superior for CAIX imaging in renal cell carcinoma xenografts, because of its high tumor to background ratios at early time points^24^. However, in the head and neck cancer models [^111^In]In-DTPA-girentuximab seems to be the best candidate, due to its high tumor uptake and normal tissue contrast with the drawback that imaging is only possible 72 hours post tracer administration. It also has to be noted that as a predictive tool in locoregional head and neck cancer the contrast between tumor and organs, such as kidneys and liver is less relevant, since the primary tumor is not in the vicinity of these organs. Therefore, the [^111^In]In-DTPA-girentuximab-F(ab′)_2_ could still be favorable in situations where fast imaging is needed.

In this study we showed that different CAIX-targeting imaging compounds are able to distinguish a low CAIX-expressing tumor from a high CAIX-expressing tumor in head and neck cancer xenografts models. In these hypoxic head and neck xenograft models the IgG based tracer, [^111^In]In-DTPA-girentuximab, showed the most promising results. Furthermore, big differences in tracer uptake in these xenografts were seen compared to studies using ccRCC models. This raises the question how these tracers would perform in patients with a hypoxic tumor.

In clinical practice tracer uptake is affected by multiple factors such as blood clearance, which are different in mice and humans and therefore these compounds might behave different in patients. Girentuximab IgG based tracers have been studied in renal cancer patients and proofed to be safe and valuable^36,37^. Until the present day, CAIX imaging has not been used routinely for the management of head and neck cancer patients. A clinical study using a girentuximab IgG based tracer could provide interesting insights in the feasibility and value of CAIX imaging in this patient group.

## Methods

### Protein production

Affibody molecule HE_3_-ZCAIX:2 was produced and conjugated with DOTA chelator as described by Garousi *et al*.^23^. Girentuximab-(Fab′)_2_ was produced by enzymatic digestion of the monoclonal chimeric anti-CAIX antibody girentuximab (G250 Wilex AG) as described by Huizing *et al*.^33^.

cG250 and cG250-F(ab′)_2_ fragments were conjugated with isothiocyanatobenzyl-diethylenetriaminepentaacetic acid (ITC-DTPA, Macrocyclis, Houston, TX, USA) in 0.1 M NaHCO_3_, pH 9.5, for 1 hour at room temperature using a ten-fold molar excess. Unconjugated ITC-DTPA was removed by extensive dialysis against 0.25 M ammonium acetate buffer, pH 5.4 using a Slide-a-Lyzer dialysis cassette (Thermo, Ma, USA).

### Radiolabeling

Labeling of DOTA-HE_3_-ZCAIX:2 with ^111^In was performed as described by Garousi *et al*.^24,38^. Briefly, 50 µg DOTA-HE_3_-ZCAIX:2 in 75 µL 0.2 M ammonium acetate, pH 5.5, was mixed with 53 MBq (50 µl) [^111^In]InCl_3_ (Petten, The Netherlands). The mixture was incubated for 60 min at 95 °C. Thereafter, a 5,000-fold excess of tetrasodium salt of ethylenediaminetetracetic acid (Na_4_EDTA) was added to the mixture. Labeling efficiency was determined by instant thin-layer chromatography (ITLC) on chromatography paper impregnated with a silica gel (Agilent Technologies), using 0.1 M citrate buffer, pH 6.0 as a mobile phase. Mean labeling efficiency was 91% (Supplementary figure 2), [^111^In]In-DOTA-HE_3_-ZCAIX:2 was purified using NAP-5 column (GE Healthcare, Uppsala, Sweden) pre-equilibrated and eluted with phosphate-buffered saline (PBS).

DTPA-conjugated cG250 and cG250-F(ab′)_2_ fragments were radiolabeled with [^111^In]InCl_3_ by adding a two-fold volume of 0.5 M 2-(N-morpholino)ethanesulfonic acid (MES), pH 5.5, buffer in relation to the volume of [^111^In]InCl_3_. Mean labeling efficiency was 92% for ^111^In]In-DTPA-cG250 and 67% for [^111^In]In-DTPA-cG250-F(ab′)_2_ (Supplementary figure 2), the radiotracer was purified on a PD-10 column (GE, Woerden, The Netherlands) pre-equilibrated and eluted with phosphate-buffered saline containing 0.5% bovine serum albumin (PBS-BSA).

Radiochemical purity of radiolabeled products exceeded 98% in all experiments and the specific activity of the preparations was 7.5 MBq/µg ([^111^In]In-DOTA-HE_3_-ZCAIX:2), 1.5 MBq/µg ([^111^In]In-DTPA-cG250-F(ab′)_2_) and 1.2 MBq/µg ([^111^In]In-DTPA-cG250).

### Xenograft tumor models

Six to eight weeks old female BALB/c nude mice (Janvier Labs, Le Genest-Saint-Ile, France) were used in all *in vivo* experiments. Starting weights of the animals was between 18 and 22 grams. After tumor growth, animals were distributed over the experimental groups. Stratified randomization was used based on tumor size, in order to have a similar mean tumor size per study group. Animals were housed together in pathogen-free filter-topped cages. The studies were approved by the Central Authority for Scientific Procedures on Animals (RU-DEC-2016-053) and carried out under supervision of the local Animal Welfare Body.

Tumor models SCCNij153 and SCCNij185 originated from biopsies taken from head and neck cancer patients at the Radboud University Medical Center^39^. An earlier study demonstrated that SCCNij153 expresses high levels of CAIX while SCCNij185 expresses very low levels of CAIX^33^. Tumor pieces of 2 mm^3^ were implanted on the right hind leg of the mice. These tumor models were used for biodistribution and microSPECT imaging. For the purpose of immunohistochemical analysis, 2 × 10^6^ SK-RC-52 cells, were injected on the right hind leg for the growth of CAIX-positive renal tumors. Mice were injected intravenously with the radiolabeled preparations when tumors reached a diameter of 6–8 mm.

### Biodistribution and quantitative microSPECT imaging

The biodistribution of three tracers ([^111^In]In-DOTA-HE_3_-ZCAIX:2, [^111^In]In-DTPA-girentuximab-F(ab′)_2_, [^111^In]In-DTPA-girentuximab) was tested in high (SCCNij153) and low (SCCNij185) CAIX-expressing xenograft models (6 groups, n = 4 mice/group). For each tracer, time point with optimal tumor uptake and tumor-to-normal tissue contrast was derived from literature: 72 h for [^111^In]In-DTPA-girentuximab, 24 h for [^111^In]In-DTPA-girentuximab-F(ab′)_2_ and 4 h for [^111^In]In-DOTA-HE_3_-ZCAIX:2^19,24,25^. These time points were selected for microSPECT/CT imaging and *ex vivo* biodistribution studies. For the head-to-head comparison of the three radiotracers, equivalent protein amounts (91 pmol) were used for [^111^In]In-DTPA-girentuximab-F(ab′)_2_ and [^111^In]In-DTPA-girentuximab. A higher amount (237 nmol) of protein was used for ^111^In]In-DOTA-HE_3_-ZCAIX:2 in order to radiolabel the protein with sufficient activity. The injected protein mass per mouse was 14 μg [^111^In]In-DTPA-girentuximab, 10 μg [^111^In]In-DTPA-girentuximab-F(ab′)_2_, and 2 μg [^111^In]In-DOTA-HE_3_-ZCAIX:2. Average injected activity was 11 ± 2.5 Mbq per mouse.

Mice were scanned with microSPECT/CT scanner (U-SPECT II, MILabs, Netherlands) in a prone position under general anesthesia (isoflurane/N_2_O) using the 1.0-mm-diameter multipinhole mouse collimator. SPECT scans were acquired in 45 min with 12 bed positions, followed by 180 second CT scans (615 µA, 65 kV). 50 minutes before euthanasia all mice were injected with pimonidazole 80 mg/kg i.p. (J.A. Raleigh Department of Radiation Oncology, University of North Carolina, USA) for hypoxia and 1 minute before euthanasia with Hoechst 33342 15 mg/kg (Sigma, Zwijndrecht, The Netherlands) i.v.

From all mice, tumors and tissue samples (blood, skin, muscle, small intestine, lung, heart, kidney, and liver) were harvested and weighed. Subsequently, radioactivity uptake was determined in a γ-counter (2480 Wizard 3″, LKB/Wallace, Perkin-Elmer,Boston, MA). Activity concentrations in the tissues were calculated as percentage of the injected dose per gram of tissue (%ID/g). To correct for radioactive decay, injection standards were counted simultaneously.

Scans were reconstructed with MILabs reconstruction software, using an ordered-expectation maximization algorithm with a voxel size of 0.375 mm. Tumor-to-muscle mean pixel value ratios were determined by drawing volumes of interest (VOIs) around the tumor and in the muscle of the hind leg. %ID/ml was calculated per VOI with the use of a standard series with known radioactivity concentrations measured with the same device and settings. (Inveon Research Workplace software, version 3.0; Siemens Preclinical Solutions).

### Immunohistochemistry and autoradiography

Immunohistochemical staining was performed following the same protocols as described by Huizing *et al*.^19^. Briefly, half of each tumor was used for biodistribution measurements while the other half was snap frozen directly after harvesting, cut into sections of 5 μm, mounted on poly-l-lysine coated slides and stored at −80 °C. Sections were fixed with acetone for 10 min at 4 °C. The intratumoral distribution of the radiotracers was determined by autoradiography. Tumor sections were exposed to a Fujifilm BAS cassette 2025 overnight (Fuji Photo Film). Phospholuminescence plates were scanned using a Fuji BAS-1800 II bioimaging analyzer at a pixel size of 25 × 25 μm. Autoradiography images were analyzed with Aida Image Analyzer software (Raytest). After autoradiography, the same slides were scanned (Axio Scope A1 (Zeiss), Coolsnap HQ2 (Hamamatsu Photonics), MAC 6000 system (Ludl Electronic Products Ltd) for Hoechst followed by immunohistochemistry staining and scanning for CAIX, pimonidazole and vessel visualization. Slides were stained successively with: rabbit anti CA9_Biotin 1:500 in primary antibody diluent (PAD) (30 min), goat anti rabbitFabCy3 1:400 in PAD (30 min), donkey anti goatCy3 1:400 in PAD (45 min), 9F1 (45 min), rabbit anti pimonidazole 1:1000 in PAD (45 min), donkey anti rabbitAlexa488 1:600 + chicken anti ratAlexa647 1:100 in PAD (30 min). All steps were performed at 37 °C and between steps slides were rinsed 3 times with PBS. Afterwards they were mounted with fluoromount.

### Intratumoral correlation of autoradiography and immunohistochemistry

To correlate spatial distribution of the radiotracer with the intratumoral CAIX expression, autoradiography images and immunohistochemistry images were overlaid. Autoradiography images were inverted and immunohistochemistry images were rescaled to match the autoradiography (25 × 25 μm per pixel) using ImageJ. To select only viable tumor area, a layer was drawn to exclude non-tumor tissue area from the image.

Images were registered using iVision software (BioVision technologies, USA). Subsequently, a parametric mapping technique was applied to reduce the spatial information in the images^40^. Hereto, all grayscale images were subdivided in squares of 10 × 10 pixels, corresponding to a size of 180 × 180 μm. Gray-values within these co-registered squares were analyzed as continuous values for spatial correlation between the imaging modalities. Spearman-test was used to test for correlation.

### Statistical analysis

Statistical analysis were performed using GraphPad Prism (version 6.0e). Descriptive data were stated as means with standard deviations. The unpaired t-test and one-way ANOVA were used to compare groups and multiple groups. Pearson correlation coefficients were used to assess correlation between autoradiography images and immunohistochemistry images.

### Ethical statement

All experiments and methods were performed in accordance with relevant guidelines and regulations. All experimental protocols were approved by a named institutional/licencing committee. Specifically, animal studies were approved by the Dutch Central Authority for Scientific Procedures on Animals (RU-DEC-2016-053) and carried out under supervision of the local Animal Welfare Body.

## Supplementary information

Supplementary data

## Data Availability

The datasets generated during and/or analyzed during the current study are available from the corresponding author on reasonable request.
