# Supplementary material for: CAIX-targeting radiotracers for hypoxia imaging in head and neck cancer models
Source: Sci Rep. 2019 Dec 11;9:18898. doi: 10.1038/s41598-019-54824-5 (PMC6906415; doi:10.1038/s41598-019-54824-5)

**Supplementary information**

Manuscript title: *CAIX-targeting radiotracers for hypoxia imaging in head and neck cancer models*

**Authors:** Fokko J Huizing^1^, Javad Garousi^2^, Jasper Lok^1^, Gerben Franssen^3^, Bianca AW Hoeben^1^, Fredrik Y Frejd^2,4^, Otto C Boerman^3^, Johan Bussink^1^, Vladimir Tolmachev^2^, Sandra Heskamp^3^

Supplementary table 1.

Biodistribution data of [^111^In]In-DOTA-HE_3_-ZCAIX:2 4h p.i.

| **SCCNij153 SCCNij185**   \| Tissue \| Mean(%ID/g) \| \| SD(%ID/g) \| \| N \| Mean(%ID/g) \| SD(%ID/g) \| \| N \| \| \| --- \| --- \| --- \| --- \| --- \| --- \| --- \| --- \| --- \| --- \| --- \| \| Blood \| 0.0651378 \| 0.03851578 \| \| 4 \| \| 0.06189735 \| \| 0.01458324 \| \| 4 \| \| Salivary glands \| 0.3804742 \| 0.03642972 \| \| 4 \| \| 0.3905837 \| \| 0.0956353 \| \| 4 \| \| Lung \| 0.2368805 \| 0.03174399 \| \| 4 \| \| 0.2885302 \| \| 0.05026733 \| \| 4 \| \| Liver \| 0.2559889 \| 0.02583422 \| \| 4 \| \| 0.2851363 \| \| 0.07446552 \| \| 4 \| \| Spleen \| 0.2652014 \| 0.04878566 \| \| 4 \| \| 0.227976 \| \| 0.05166009 \| \| 4 \| \| Stomach \| 0.2067512 \| 0.02865861 \| \| 4 \| \| 0.2133583 \| \| 0.0260585 \| \| 4 \| \| duodenum \| 0.1778417 \| 0.02028324 \| \| 4 \| \| 0.1800843 \| \| 0.05776634 \| \| 4 \| \| colon \| 0.2220501 \| 0.08761856 \| \| 4 \| \| 0.2181757 \| \| 0.0592407 \| \| 4 \| \| Kidney \| 188.4234 \| 16.57659 \| \| 4 \| \| 189.9241 \| \| 26.66915 \| \| 4 \| \| tumor \| 0.3240742 \| 0.0564798 \| \| 4 \| \| 0.1837626 \| \| 0.01932129 \| \| 4 \| \| muscle \| 0.1116655 \| 0.04692388 \| \| 4 \| \| 0.2371295 \| \| 0.2202472 \| \| 4 \| \| bone \| 0.1276376 \| 0.04864071 \| \| 4 \| \| 0.1416588 \| \| 0.03405686 \| \| 4 \| \| GI tract \| 0.508677 \| 0.639853 \| \| 4 \| \| 0.2793746 \| \| 0.1152066 \| \| 4 \| \| tail \| 0.1752128 \| 0.04733546 \| \| 4 \| \| 0.5448871 \| \| 0.5437524 \| \| 4 \| |  |  |
| --- | --- | --- | --- | --- | --- | --- | --- | --- | --- | --- | --- | --- | --- | --- | --- | --- | --- | --- | --- | --- | --- | --- | --- | --- | --- | --- | --- | --- | --- | --- | --- | --- | --- | --- | --- | --- | --- | --- | --- | --- | --- | --- | --- | --- | --- | --- | --- | --- | --- | --- | --- | --- | --- | --- | --- | --- | --- | --- | --- | --- | --- | --- | --- | --- | --- | --- | --- | --- | --- | --- | --- | --- | --- | --- | --- | --- | --- | --- | --- | --- | --- | --- | --- | --- | --- | --- | --- | --- | --- | --- | --- | --- | --- | --- | --- | --- | --- | --- | --- | --- | --- | --- | --- | --- | --- | --- | --- | --- | --- | --- | --- | --- | --- | --- | --- | --- | --- | --- | --- | --- | --- | --- | --- | --- | --- | --- | --- | --- | --- | --- | --- | --- | --- | --- | --- | --- | --- | --- | --- | --- | --- | --- | --- | --- | --- | --- | --- | --- | --- | --- | --- | --- | --- | --- | --- | --- | --- | --- | --- | --- | --- | --- | --- | --- | --- | --- | --- |
|  |  |  |

Supplementary table 2.

Biodistribution data of [^111^In]In-DTPA-cG250-(Fab’)_2_ 24h p.i.

**SCCNij153 SCCNij185**

| Tissue | Mean(%ID/g) | | SD(%ID/g) | | N | Mean(%ID/g) | SD(%ID/g) | | N | |
| --- | --- | --- | --- | --- | --- | --- | --- | --- | --- | --- |
| Blood | 0.2536704 | 0.1274991 | | 3 | | 0.1414372 | | 0.02107245 | | 3 |
| salivary glands | 0.6741324 | 0.0851181 | | 3 | | 0.9013447 | | 0.2691289 | | 3 |
| Lung | 0.6568447 | 0.07058452 | | 3 | | 0.8673878 | | 0.1103974 | | 3 |
| Liver | 4.687017 | 1.217335 | | 3 | | 6.709292 | | 1.284829 | | 3 |
| Spleen | 6.871529 | 2.099776 | | 3 | | 7.57554 | | 1.536436 | | 3 |
| Stomach | 0.7570792 | 0.1655017 | | 3 | | 0.7050366 | | 0.09772237 | | 3 |
| duodenum | 0.9536418 | 0.1099314 | | 3 | | 1.132837 | | 0.07030472 | | 3 |
| colon | 0.9182714 | 0.1917884 | | 3 | | 0.8540085 | | 0.05954214 | | 3 |
| Kidney | 71.47349 | 4.886881 | | 3 | | 107.5551 | | 8.128808 | | 3 |
| tumor | 3.056882 | 1.175911 | | 3 | | 1.236267 | | 0.1408671 | | 3 |
| muscle | 0.2963944 | 0.2208789 | | 3 | | 0.5194938 | | 0.4763279 | | 3 |
| bone | 1.084702 | 0.418383 | | 3 | | 1.150307 | | 0.2048742 | | 3 |
| GI tract | 0.8917675 | 0.2216521 | | 3 | | 1.166606 | | 0.4961318 | | 3 |
| tail | 1.093436 | 0.4148805 | | 3 | | 2.410962 | | 0.4475081 | | 3 |

Supplementary table 3.

Biodistribution data of [^111^In]In-DTPA-cG250 72h p.i.

**SCCNij153 SCCNij185**

| Tissue | Mean(%ID/g) | | SD(%ID/g) | | N | Mean(%ID/g) | SD(%ID/g) | | N | |
| --- | --- | --- | --- | --- | --- | --- | --- | --- | --- | --- |
| Blood | 12.65301 | 3.838871 | | 5 | | 8.418246 | | 1.476941 | | 3 |
| salivary glands | 4.463443 | 0.7836447 | | 5 | | 5.088856 | | 1.318051 | | 3 |
| Lung | 5.856074 | 1.6369 | | 5 | | 4.452848 | | 0.5159787 | | 3 |
| Liver | 6.342065 | 0.08283318 | | 5 | | 6.218151 | | 2.043484 | | 3 |
| Spleen | 7.002428 | 1.479345 | | 5 | | 5.954615 | | 1.223489 | | 3 |
| Stomach | 1.907595 | 0.263288 | | 5 | | 1.650485 | | 0.03437597 | | 3 |
| duodenum | 1.909452 | 0.3355312 | | 5 | | 1.97863 | | 0.2385516 | | 3 |
| colon | 1.311759 | 0.1477102 | | 5 | | 1.418511 | | 0.2328355 | | 3 |
| Kidney | 7.225246 | 0.7987807 | | 5 | | 6.928051 | | 1.216905 | | 3 |
| tumor | 30.3646 | 4.593 | | 5 | | 6.992333 | | 1.708357 | | 3 |
| muscle | 1.177323 | 0.07133935 | | 5 | | 0.9329388 | | 0.1825535 | | 3 |
| bone | 2.158064 | 0.2247012 | | 5 | | 1.335469 | | 0.2629641 | | 3 |
| GI tract | 6.850879 | 11.37474 | | 5 | | 1.600483 | | 0.02519431 | | 3 |
| tail | 2.494606 | 0.1792261 | | 5 | | 2.000704 | | 0.1682154 | | 3 |

Supplementary figure 1.

Correlation of *ex vivo* tumor uptake measurements with *in vivo* SPECT measurements per tumor R = 0.92 (p=0.001).


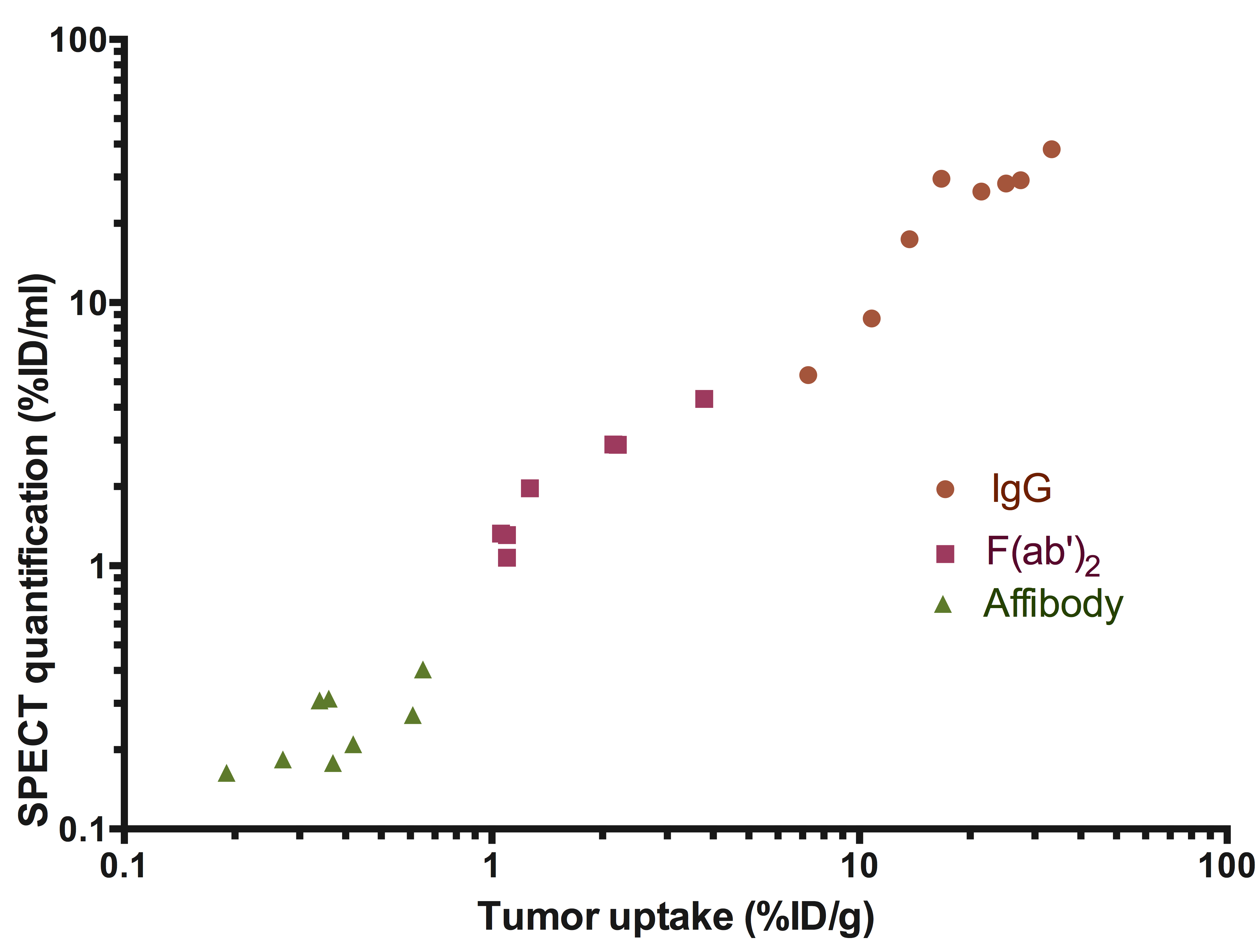


Supplementary figure 2.

Radiolabeling [^111^In]In- DOTA-HE_3_-ZCAIX:2, ITLC measurements before (A) and after (B) purification.


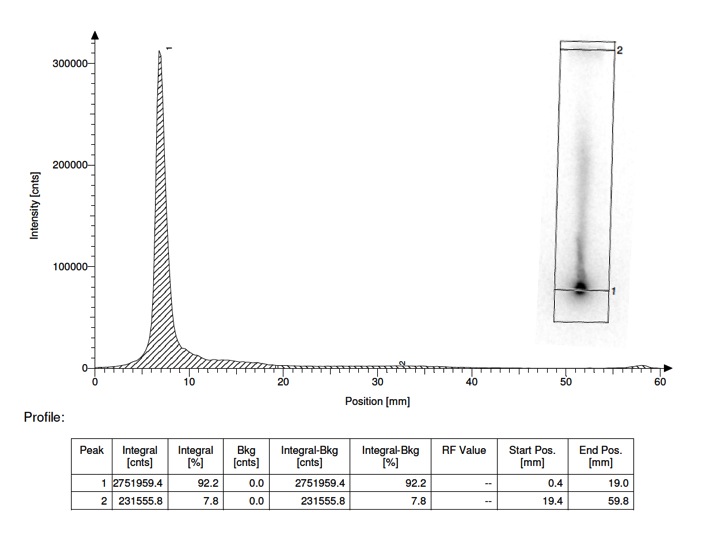

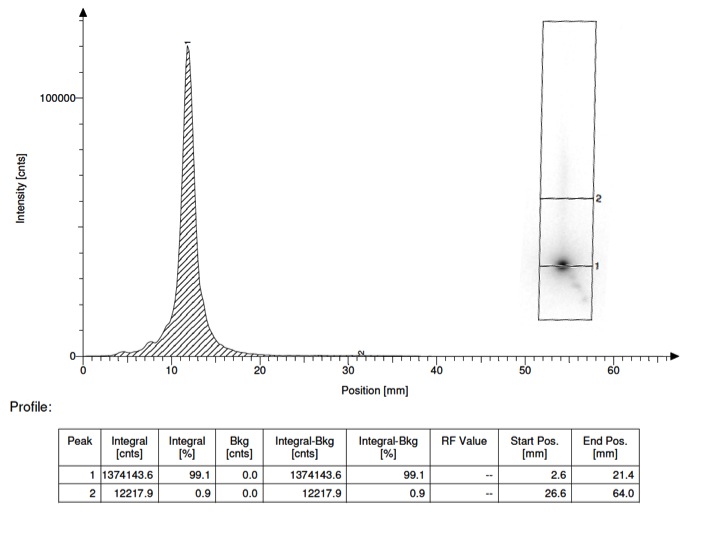
A B


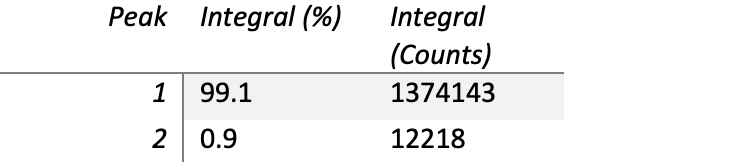

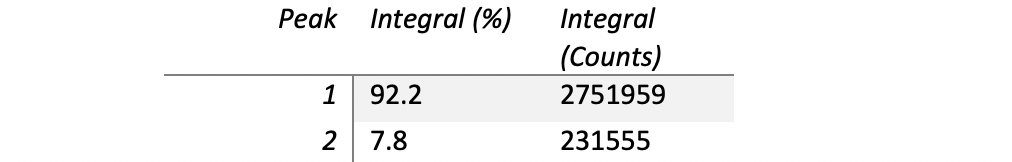


Supplementary figure 3.

Radiolabeling [^111^In]In-DTPA-cG250-(Fab’)_2_, ITLC measurements before (A) and after (B) purification.


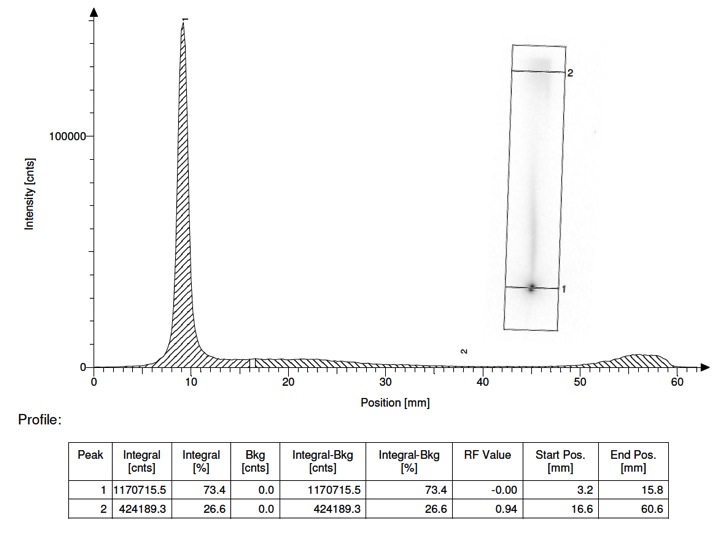
A B


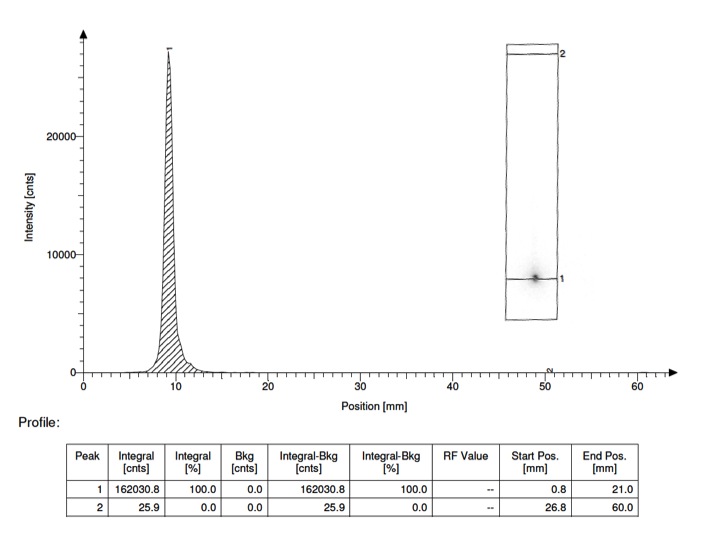


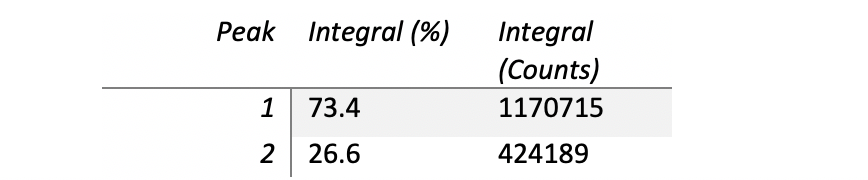


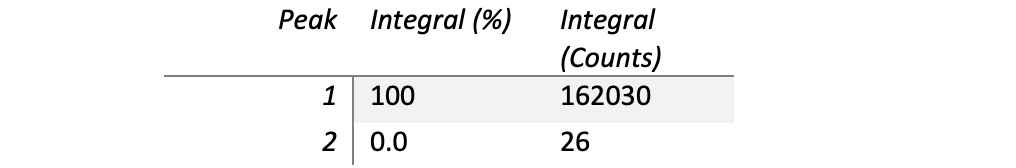


Supplementary figure 4.

Radiolabeling [^111^In]In-DTPA-cG250, ITLC measurements before (A) and after (B) purification.


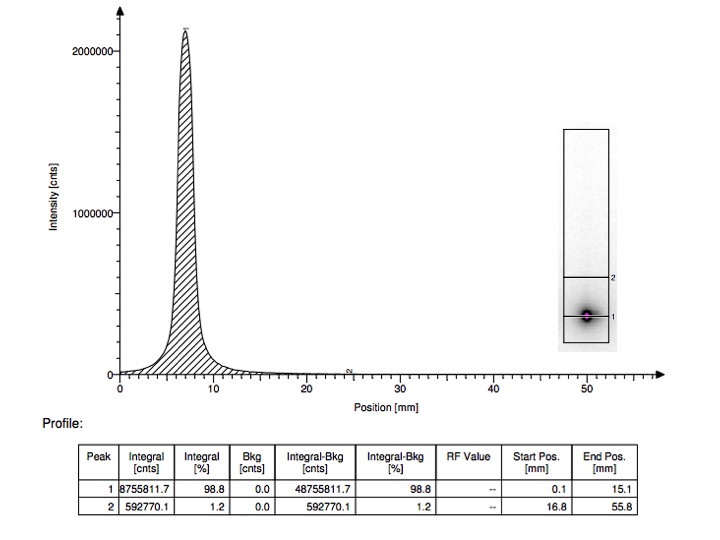

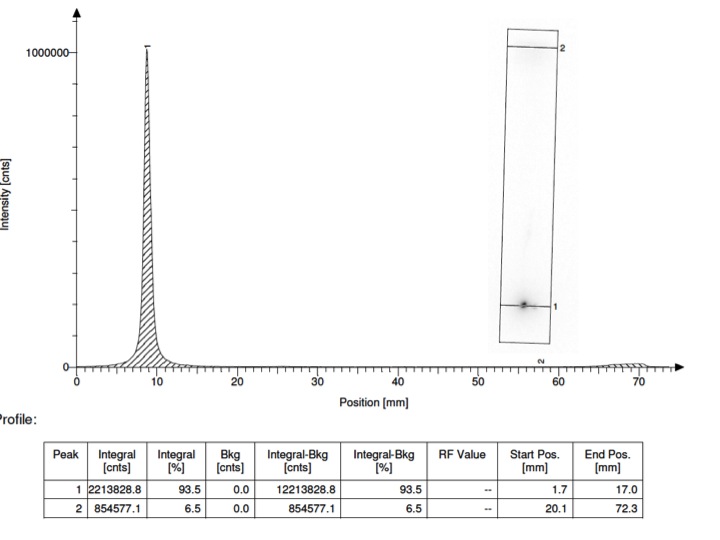
A B


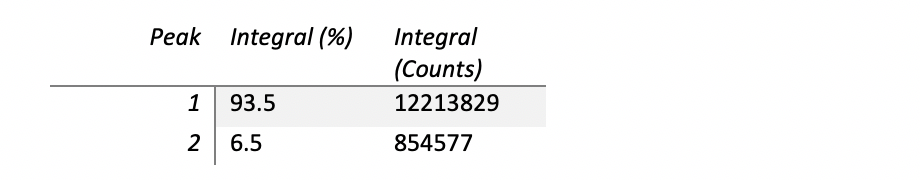

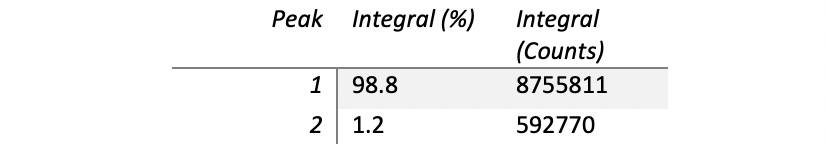

Supplement: Supplementary file 1 — Supplementary data [file 41598_2019_54824_MOESM1_ESM.docx]
